# The role of hsa_circ_0008945 in juvenile-onset systemic lupus erythematosus

**DOI:** 10.1186/s12920-023-01524-9

**Published:** 2023-05-09

**Authors:** Qifan Wang, Baiye Xu, Qingmei Zhang, Haidao Wang, Shulian Chen, Tingting Chen, Shishan Liang

**Affiliations:** Department of Pediatrics, The First Hospital of Quanzhou Affiliated to Fujian Medical University, Quanzhou, 362000 Fujian PR China

**Keywords:** Juvenile-onset systemic lupus erythematosus, CircRNA, Circ_0008945, Biomarker, Apoptosis

## Abstract

**Background:**

circular RNAs (circRNAs) play a crucial role in many physiological and pathological processes including juvenile-onset systemic lupus erythematosus (JSLE). The aim of this study is to investigate the role of circRNA hsa_circ_0008945 in JSLE and evaluate its significance as diagnosing biomarker.

**Methods:**

RT-qPCR was applied to detect the level of circ_0008945 in JSLE and controls. The Spearman correlation test assessed the correlation between circ_0008945 and clinical variables. The receiver operating characteristic (ROC) curve was calculated for evaluating the diagnostic value. Overexpression or knockdown of circ_0008945 in primary peripheral blood mononuclear cells (PBMCs) was performed to further examine its function in apoptosis.

**Results:**

RT-qPCR revealed that there were significantly higher levels of hsa_circ_0008945 in PMBCs from JSLE patients (p < 0.001) compared to healthy controls. In addition, there were significant associations between hsa_circ_0008945 level and the level of C3, C4, anti-ds DNA, IgG, CRP and ESR (p < 0.05) but not associated with the level of Ig A and Ig M. ROC curve of the circ_0008945 showed that the AUC was 0.790 and it may potentially be used as a novel biomarker for the diagnosis of JSLE. The results showed that overexpression of circ-0008945 increased the apoptosis of PBMCs while knockdown of circ-0008945 by siRNA decreased the apoptosis of PBMCs, supporting that circ-0008945 promoted the apoptosis in PBMCs and contributed to the pathogenesis of JSLE.

**Conclusion:**

The role of circ_0008945 was first investigated in JSLE and proposed herein their possible contribution to the pathogenesis of JSLE. This study provides not only novel insight into the pathological mechanisms but also the potential value as a useful biomarker for JSLE.

## Introduction

Systemic lupus erythematosus (SLE) belongs to an autoimmune disease in which itstissues are heavily attacked by the dysfunctional immune system [1]. The misregulation of T and B lymphocytes and other cells in the immune system are the main actors involved in the pathogenesis of SLE. They produce many autoantibodies and other inflammatory cytokines that damaged tissues and organs and lead to a variety of clinical [2]. Juvenile-onset systemic lupus erythematosus (JSLE) onset before 16-year-old and accounted for approximately 15–20% of total SLE patients. JSLE normally is more aggressive than adult SLE with increased morbidity and [3, 4]. The diversity and complexity of clinical manifestations in some diseases like JSLE bring some difficult and differential diagnoses. Therefore, it is of great significance to find specific and sensitive biomarkers for JSLE and explore its pathogenic mechanism of JSLE.

circRNAs are produced by non-canonical back-splicing events with covalently closed loop [5] and are more difficultly degraded by [5]. The main function of circRNAs includes microRNA (miRNA) sequestration, modulating protein–protein interactions and mRNA transcription regulation. The main described function of the circRNAs is acting as ‘miRNAs sponges’ [5, 6]. Considerable studies have revealed that circRNA plays critical roles in gene expression and regulating various immune [6, 7]. For instance, It was reported that exosomal circRNA-002178 induced the PD1 expression in CD8 T cells and resulted in T cell [8]. The study demonstrated that circSnx5 triggered dendritic cells (DC) activation and therefore regulated DC-driven immunity and tolerance via the circSnx5/miR-544/SOCS1 [9]. Studies also showed that circRNA was involved in immune related diseases including rheumatoid arthritis [10] and adult SLE [11–13]. CircRNAs can distribute in membrane-bound vesicles such as exosome and body fluids including urine and blood [8]. Therefore, circRNA can be served as biomarkers for clinical diagnosis of diseases including autoimmune diseases. Notedly there was evidence supporting that the non-coding RNAs (ncRNAs) including circular RNAs (circRNAs) contributed to the pathogenesis and progression of [10, 11]. A recent study demonstrated that the level of hsa_circ_0000479 increased in PBMCs of SLE patients and hsa_circ_0000479 might be served as a potential biomarker for SLE diagnosis and therapeutic [12]. Compared to adult SLE, the role of ncRNA including circular RNA is little investigated in JSLE. Recently a research team screened and analyzed the circRNA expression profile in [13] and indicated the level of the circRNA hsa_circ_0008945 was significantly higher in JSLE than in controls. However, the role of circRNA in JSLE has not explored yet. This study is to investigate the role of circRNA hsa_circ_0008945 in JSLE. This study showed that there was significantly higher levels of hsa_circ_0008945 in PMBCs from JSLE patients compared to controls. circ_0008945 may act as a potential biomarker for the diagnosis of JSLE and contribute to the pathogenesis of JSLE.

## Materials and methods

### Subject

A total of 30 JSLE participants (under 16-year-old) and 30 age and sex-match healthy controls were consecutively recruited for this study from the First Hospital of Quanzhou Affiliated to Fujian Medical University from Sep 2019 to Aug 2020. The JSLE patients were those less than 16-year-old at the onset of the disease. All JSLE patients were ethnic Chinese and fulfilled the revised criteria of SLE in the American College of Rheumatology (ACR) 1997 [22]. Disease activity was assessed following the 2000 SLE Disease Activity Index (SLEDAI) [25]. All study protocols were approved by the Ethics Committee of the First Hospital of Quanzhou Affiliated to Fujian Medical University [Approved No: Quan-Lun (2019)156]. All the legal guardians of the participants were informed and provided written informed consents.

### Collection of PBMCs and total RNA extraction

6ml Blood was collected with EDTA per participant and peripheral blood mononuclear cells (PBMCs) were isolated by the Ficoll-Histopaque density gradient centrifugation method. Samples were cryopreserved and stored at − 80 °C within 6 h after collection. At the same time, blood samples from JSLE patients were collected for routine blood tests including and measuring C-reactive protein, complement C3, C4, anti-dsDNA, IgG, IgA, IgM, ESR, etc. Total RNA extraction was isolated from PBMCs with Neurozol reagent (MACHEREY-NAGEL, Germany) according to the manufacturer’s instructions. The concentrations of the RNA were determined by OD260 using a NanoDrop ND-1000 (Thermo Fisher Scientific, USA).

### RT-qPCR

Total RNA was extracted using Neurozol reagent (MACHEREY-NAGEL, Germany) and reverse transcription reagent kit (PROMEGA, USA) was used for generating cDNA. SYBR Green PCR kit (Takara, China) was employed for running real-time PCR. GAPDH were internal controls. The qPCR analysis was performed on an ABI 7500 Real-time PCR System (Applied Biosystems, Thermo Fisher Scientific, USA) according to the instructions supplied by the manufacturer. The relative expression levels of the genes were calculated by comparing GAPDH using 2 − ΔΔCT method. The primers were used as follows:

circ_0008945 FOR CTGACCTACGAGTGACTCAGC;

circ_0008945 REV GCTGGAAGTCAGAGAGATCGG;

GAPDH FOR AGAAGGCTGGGGCTCATTTG;

GAPDH FOR AGAAGGCTGGGGCTCATTTG;

### pcDNA-circ_0008945 vectors and siRNA

The sequence of hsa_circ_0008945 (circBase ID: hsa_circ_0008945; 384 bp) was obtained from human circRNA database circBank (http://www.circbank.cn). The full-length sequence of hsa_circ_0008945 was custom synthesized and inserted between BamHI and XhoI site of pcDNA3.0 vector((Invitrogen). The pcDNA3.0 vector was employed as negative control (OE-NC). si-NC (negative control; sequence: TTCTCCGAACGTGTCACGTTT), si-circRNA (si-hsa_circ_0008945 sequence: ATGCTGGTGGCAAGCTGCACATT) were synthesized by GenePharma (Shanghai, China).

### Primary PBMCs culture and cell transfection

The collected PBMCs from above method were cultured with supplemented RPMI-1640 medium in 24-well plates and incubated at 37 °C in a humidified, 5% CO2 atmosphere. When the subcultured PBMCs in 6-well plate grew to 70–80%, cell transfection with SiRNA or pcDNA using Lipofectamine 3000 Reagent (Life Technologies, USA) was carried out and then further cultured in at 37 °C and 5% CO2 for 48–72 h.

### FACS analysis

A total number of 1 × 10E6 PMBCs were stained with Annexin V-FITC (0.5ug/ml) and propidium iodide (PI, 5ul/ml) for 10 min and processed for Flow cytometry using a FACS cytometer (FACSVerse, BD Biosciences) without delay.

### Statistical analysis

Statistical analysis was processed with GraphPad Prism version 7.0 (GraphPad Software, USA) and SPSS version 23.0 (IBM. USA). A Student’s t-test or nonparametric Mann–Whitney test was applied to analyze the data. Likewise, The Pearson method or the nonparametric Spearman method was employed for correlation analysis. Receiver operating characteristic (ROC) curves were performed to evaluate the diagnostic value of circRNAs. The value of p < 0.05 is considered as significant difference.

## Results

### Basic information for the JSLE patients and controls

The age and sex of the JSLE patients and healthy controls were not significantly different. The clinical data for the patients and controls were shown in Table 1. The laboratory data of JSLE patients and Healthy controls were shown in Table 2 and the level of C3, C4, anti-ds DNA, IgG, CRP and ESR were higher in JSLE patients compared to that of controls with significant difference, but IgA and Ig M were not different.

**Table 1:** Clinical data of JSLE patients and Healthy controls

	JSLE Patients(30)	Healthy Control(30)	p-value
Age (mean ± SD)	10.133 ± 2.193	10.067 ± 2.348	0.910
Sex (female/male)	17/13	18/12	0.793
Disease duration (month)	5.667 ± 4.505	N	N
SLEDAI	7.467 ± 2.956	N	N

**Table 2:** Laboratory data of JSLE patients and Healthy controls

	Patients(30)	Control(30)	t/χ^2^		p-value
C3(g/l)	0.468 ± 0.315	1.143 ± 0.239	9.361		<0.001
C4(g/l)	0.064 ± 0.041	0.277 ± 0.066	-6.652		<0.001
anti-dsDNA	10/20	30/0	30.000		<0.001
IgG(g/l)	13.964 ± 6.956	10.267 ± 2.040	-1.811		0.047
IgA(g/l)	2.277 ± 0.756	2.524 ± 0.868	-0.251		0.802
IgM(g/l)	1.766 ± 0.740	1.938 ± 0.654	-0.961		0.336
CRP(mg/l)	8.041 ± 3.771	4.980 ± 1.566	-4.106		<0.001
ESR(mm/h)	47.000 ± 15.077	10.533 ± 2.417	-13.081		<0.001

### The level of hsa_circ_0008945 was higher in JSLE and correlated with the laboratory data

**Fig. 1:**
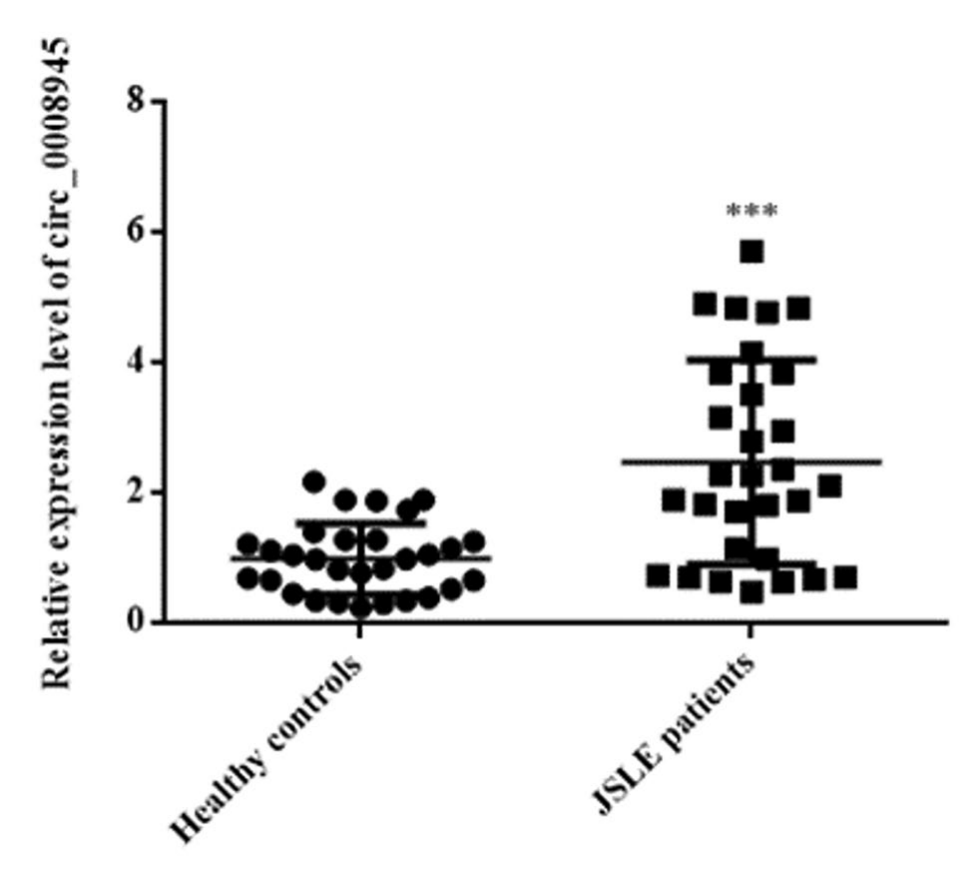
The relative expression levels of circular RNA circ_0008945 in PBMCs from JSLE patients and healthy controls was detected by RT-qPCR

**Fig. 2:**
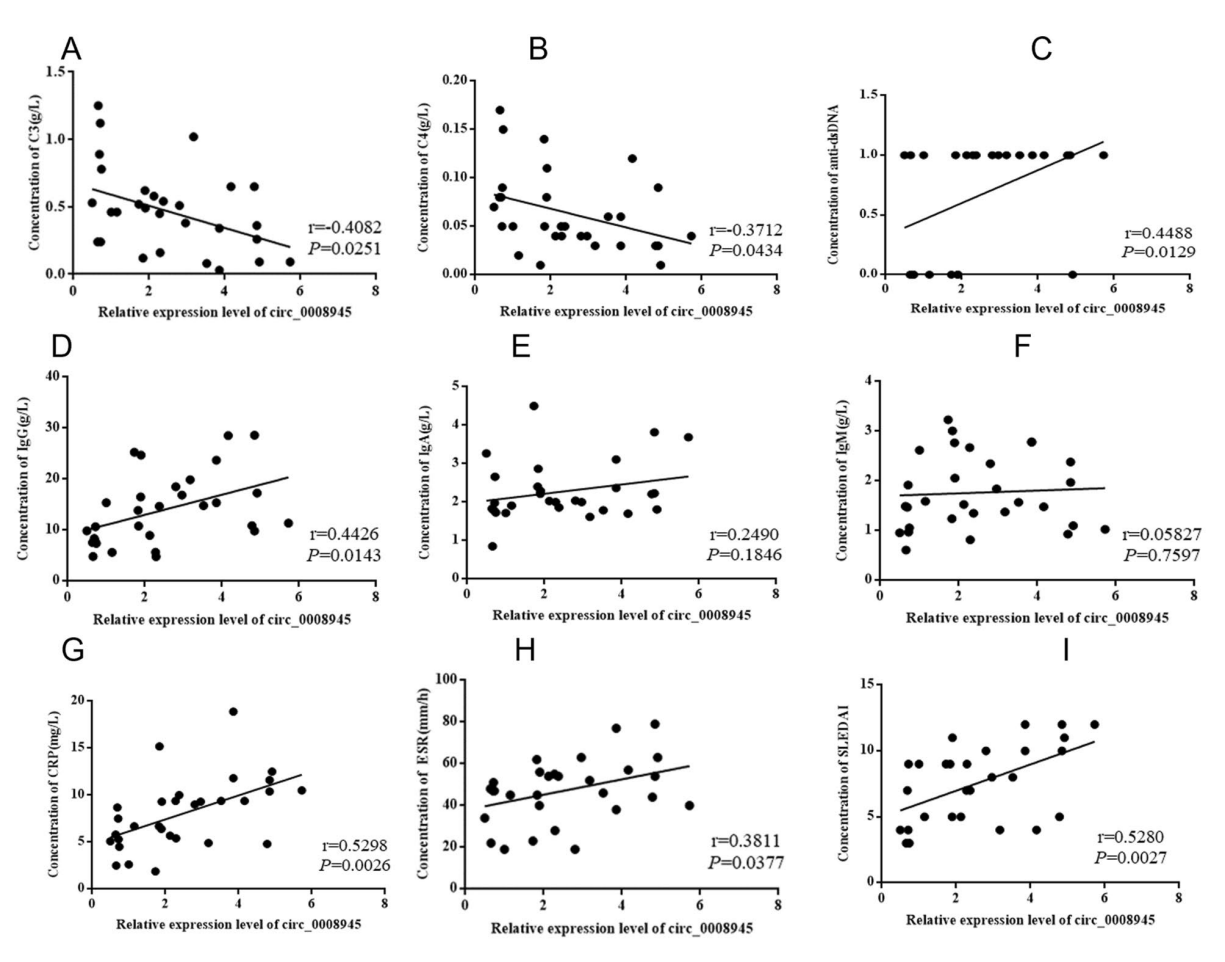
Correlation between the level of circ_0008945 in PMBCs and the laboratory data of JSLE patients. The Spearman method was performed to analyze the correlation between circ_0008945 and C3 (A), C4(B), dsDNA (C), IgG(D), CRP(H), ESR(I) and SLEDAI(J)

The expression of circ_0008945 in PMBCs from JSLE patients and controls was examined using quantitative RT-PCR and showed that there were significantly higher expression levels of hsa_circ_0008945 in PMBCs from JSLE (p < 0.001, Fig. 1). In addition, we analyzed the correlation between expressions of hsa_circ_0008945 and the clinical characteristics of JSLE patients. It showed that there was significant correlation between has_circ_0008945 level and the level of C3, C4, anti-ds DNA, Ig G, CRP and ESR (p < 0.05) but not associated with the level of Ig A and Ig M (Fig. 2).

### Potential diagnostic values of circ_0008945 in PBMCs from JSLE

**Fig. 3:**
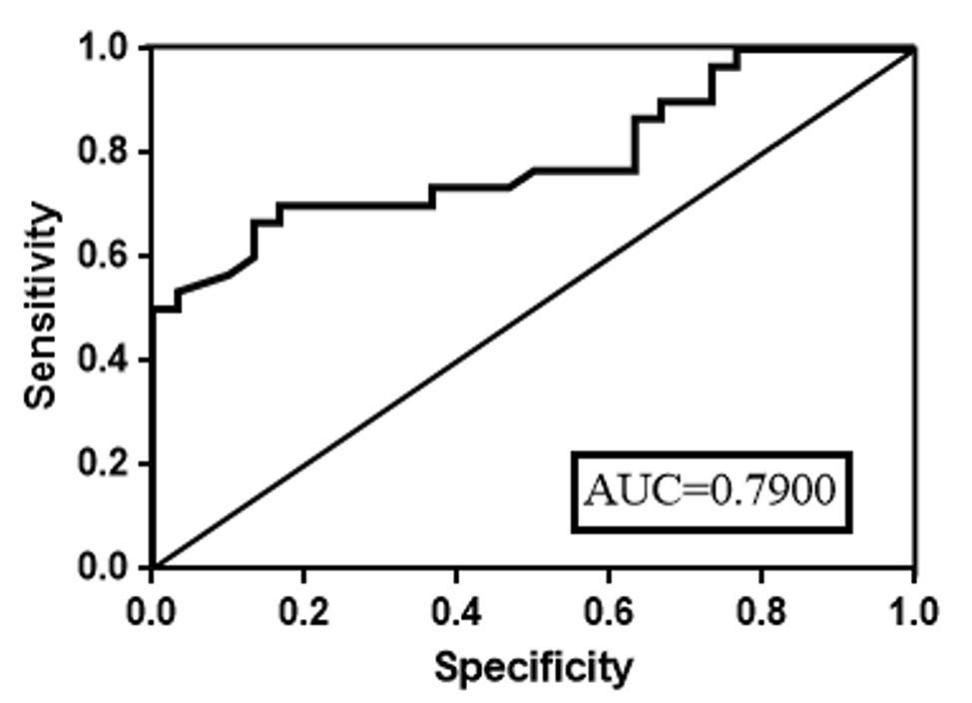
Receiver operating characteristic (ROC) curve analysis was performed to evaluate the potential diagnostic values of circ_0008945 in PBMCs from JSLE patients

Receiver operating characteristic (ROC) curve analysis was performed to evaluate the potential diagnostic values of circ_0008945 in PBMCs for JSLE. ROC curves of the circ_0008945 showed that AUC: 0.790 (95%CI: 0.6733 to 0.9067, P < 0.001; Fig. 3), Sensitivity 70%, Specificity 83.33%. These data indicated hsa_circ_0008945 in PBMCs may serve as a novel diagnostic biomarker for JSLE.

### hsa_circ_0008945 promotes apoptosis of PBMCs in JSLE

**Fig. 4:**
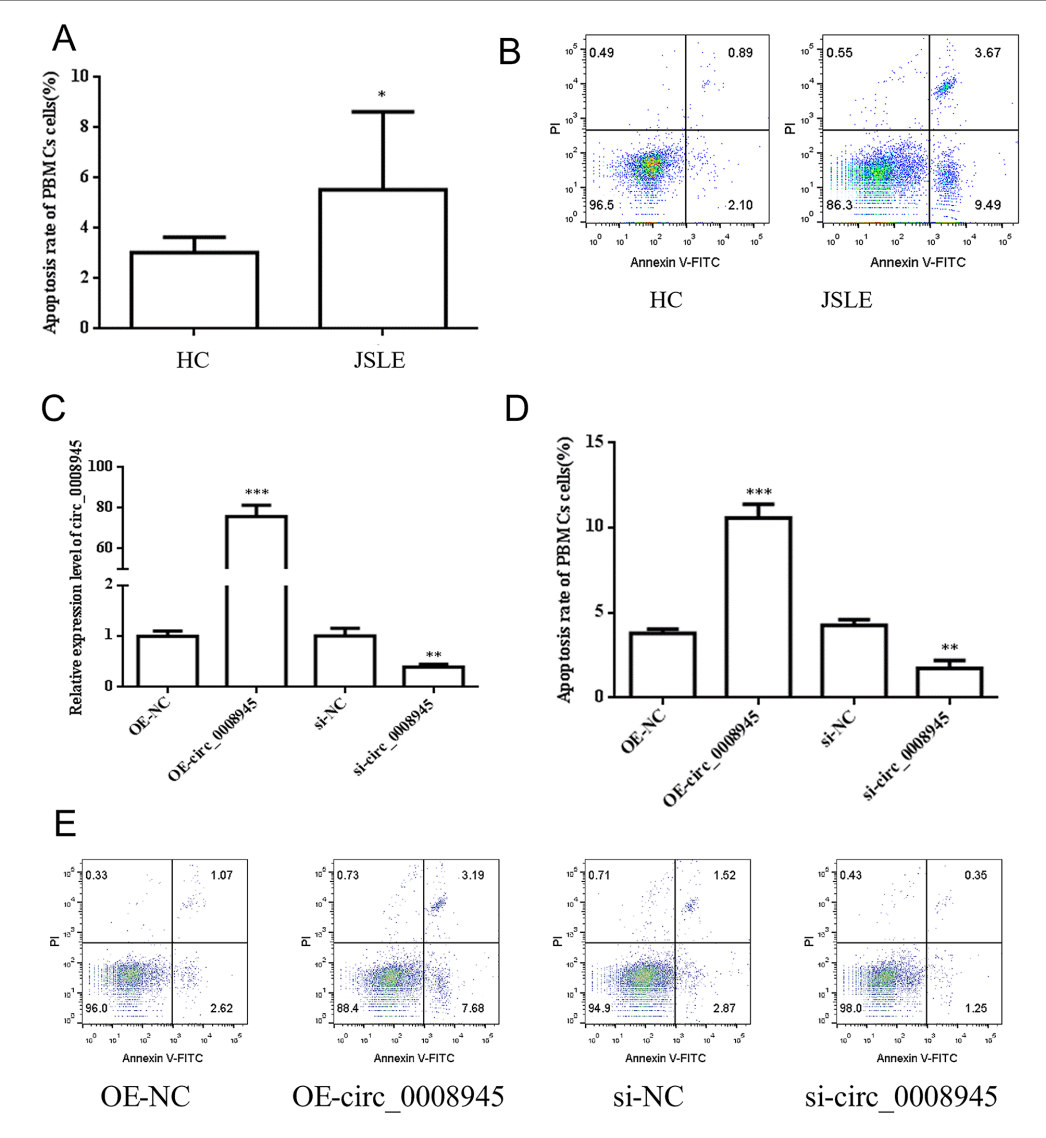
The role of circ-0008945 in apoptosis of PBMCs from JSLE. (A) Flow cytometric analysis was performed to detect the apoptosis in PBMCs from HC and JSLE. (B) Representative graphs of cell apoptosis determined by Annexin V and PI staining in PBMCs from HC and JSLE. (C) RT-qPCR was performed to check the effect of overexpression or knockdown of circ-0008945. (D) Flow cytometric analysis was performed to detect the apoptosis in PBMCs transfected with OE-NC and OE-circ-0008945 or si-NC and si-0008945. (E) Representative graphs of cell apoptosis in PBMCs transfected with OE-circ-0008945 or Si-0008945

To explore the function of hsa_circ_0008945 in PBMCs, the flow cytometric analysis was performed to detect the apoptosis of PBMCs from JSLE and controls. The results showed that the apoptosis rate of PBMCs was higher in JSLE than that of HC (Fig. 4A, B). To further investigate the role of circ-0008945 in PBMCs, we produced the plasmid vector to overexpress the circ_0008945 and si-RNA to knockdown the circ_0008945. RT-qPCR confirmed the effect of circ-0008945 overexpression with plasmid or knockdown of circ-0008945 by siRNA (Fig. 4C). Next, flow cytometric analysis was performed to detect the apoptosis in PBMCs with overexpression (OE) or knockdown of circ-0008945. The results showed that overexpression of circ-0008945 (OE-circ-0008945) increased the apoptosis of PBMCs compared to negative control while knockdown of circ-0008945 by siRNA decreased the apoptosis of PBMCs (Fig. 4D, E). All these indicated that circ-0008945 promoted apoptosis in PBMCs.

## Discussion

In this study, our data indicated that there was an increased level of has_circ_0008945 detected by RT-qPCR in JSLE patients compared to healthy controls. Correlation analysis indicated the level of circ_0008945 was positively correlated with dsDNA, CRP, ESR and SLEDA. ROC curve analysis suggested that circ_0008945 has significant diagnose value for JSLE (AUC = 0.79, p < 0.001). We also provided evidence that circ_0008945 promoted the apoptosis in PBMCs via overexpression or knockdown by siRNA targeted to circ-0008945 which contributed to the pathogenesis of JSLE.

Plenty of studies have investigated that miRNAs and lncRNAs were involved in the pathogenesis and served as potential biomarkers for [10, 11, 14]. The circRNAs are more stable than miRNAs and lncRNAs in [15], providing more potential biomarkers for human diseases. There was evidence that circRNA was involved in SLE and also served as diagnostic biomarkers. For example, a recent study validated that PBMCs hsa_circ_0000479 were increased while hsa_circ_0000175 was significantly decreased in SLE patients than in rheumatoid arthritis (RA) patients, ankylosing spondylitis (AS) patients and HC and ROC curve analysis suggested that hsa_circ_0000479 has significant value in distinguishing SLE from RA patients, AS patients, and HC (AUC = 0.825, p < 0.001) [12]. Another study indicated that the levels of hsa_circRNA_407176 and hsa_circRNA_001308 were decreased in both plasma and PBMCs of SLE compared to healthy controls. The (ROC) curve area of hsa_circRNA_407176 and hsa_circRNA_001308 were 0.599 and 0.662 in plasma, and 0.806 and 0.722 in PBMC [13]. Li et al. has demonstrated that there was dysregulation of circRNAs in PMBCs from adult SLE and hsa_circ_0045272 preventing apoptosis and interleukin-2 [16]. Compared to adult SLE, few studies for circRNA were investigated on JSLE. A recent study explored the expression of circRNAs profile in JSLE and found some misregulated circRNAs in JSLE and hsa_circ_0008945 was one of the increased circRNAs among them [17]. hsa_circ_0008945 is located in chr14:51208299–51,211,083 and its host gene is Homo sapiens ninein (NM_020921). Another study showed that circ_0008945 was increased in breast cancer(BC) and promoted BC cell proliferation, migration and invasion by sponging miR-1271 [18] and miR-338-3p to release HOXA3 [19]. However, the role and diagnostic value of hsa_circ_0008945 in JSLE is not explored yet.

Given the potential of diagnostic biomarkers, we explored the diagnostic value of circ-0008945 in PBMCs from JSLE and controls. Our study demonstrated that the expressed level of circ-0008945 in PBMCs from JSLE patients was higher than controls and it was correlated with dsDNA, C3, C4, CRP, ESR and SLEDAI, which were clinical indicators for SLE. These indicated that hsa_circ_0008945 was correlated with pathogenesis and organ damage in JSLE. Subsequently, the ROC curve analysis showed that the AUC of hsa_circ_0008945 was greater than 0.7 (0.790) and was valuable as a relevant biomarker of JSLE diagnosis and prognosis evaluation. The increased hsa_circ_0008945 in PBMCs from JSLE indicated that it might play a role on the pathogenesis of the disease, therefore, we also explored the potential pathogenic mechanism of hsa_circ_0008945 in JSLE. Our data demonstrated that circ-0008945 promoted the apoptosis of cultured PMBCs investigated by overexpression or knockdown. The circ-0008945 may contribute to the pathogenesis of JSLE via increasing apoptotic PBMCs and compromising the function of PMBCs. Increased apoptotic PBMCs from JSLE participated in the pathogenesis of the disease due to its defective/reduced function such as phagocytosis of apoptotic cells in the body [20]. Reduced clearance of other apoptotic cells by compromised PBMCs led to an increase in uncleared apoptotic cells which might act as autoantigens to trigger an autoimmune response in SLE [21–23]. Moreover, defective clearance and deposition of immune complexes (IC) can attack the tissue and lead to tissue damage in SLE [24, 25].

However, there are some limitations in this study. First, the size of the participants was comparatively small. A larger sample size is absolutely required for further investigation. Second, bioinformatics analysis has not been explored yet in the study and evidence on the molecular mechanisms/downstream targets of circ_0008945 in JSLE is currently lacking.

In summary, our study revealed that hsa_circ_0008945 was increased in PMBCs from JSLE patients and it was correlated to the level of C3, C4, anti-ds DNA, IgG, CRP and ESR. ROC curves of the circ_0008945 showed that the AUC was 0.790 and indicated that it was valuable for serving as a novel diagnostic biomarker for JSLE. We also demonstrated that circ-0008945 promoted the apoptosis in PBMCs from JSLE which may lead to defective clearance and deposition of immune complexes (IC) which can attack the tissue. This may offer new insights into the pathogenesis of JSLE. Further investigations are required to explore the molecular mechanism of circ-0008945 and the therapeutic target for JSLE.

## Data Availability

All data generated or analyzed during the current study are available from the corresponding author on reasonable request.
